# Phlebosclerotic colitis in a non-Asian patient: a case report

**DOI:** 10.1186/s13256-021-02930-2

**Published:** 2021-07-18

**Authors:** J. Mohigefer, P. Gómez-Millán, J. J. Borrero

**Affiliations:** 1 Pathology Unit, Hospital Universitario Virgen del Rocío, Seville, Spain; 2 Radiology Unit, Hospital Universitario Virgen del Rocío, Seville, Spain

**Keywords:** Phlebosclerotic colitis, Submucosal veins, Ischemic colitis, Calcification, Case report

## Abstract

**Background:**

Phlebosclerotic colitis is a rare condition with a high mortality. It has been seen almost exclusively in Asian patients who are ≥ 60 years old, with a slight male predominance. Although it predominantly affects the right colon and seems to be related in some cases to using natural herbal medicines, neither its etiology nor its pathogenesis are known.

**Case presentation:**

We present an extremely rare case of a 62-year-old Spanish white man patient of non-Asian ethnicity with no history of using natural medications, who was diagnosed with phlebosclerotic colitis of submucosal veins.

**Conclusion:**

To date, this is the only case reported in Spain, and only the second reported for Europe, in the literature. Due to the nonspecific symptoms and insidious radiological findings of this disease (both in early and mild stages) as well as exclusive submucosal involvement presented here, it is necessary that the treating physician has a high level of suspicion for its diagnosis.

## Background

Phlebosclerotic colitis is a type of ischemic colitis whose etiopathogenesis is yet to be determined [1]. It is characterized by calcification and obstruction of the submucosal and mesenteric veins of the large intestine, causing chronic ischemia. The treating physician must have a high level of suspicion, as both endoscopic and radiological findings can go unnoticed [2]. Phlebosclerotic changes are frequent in patients with venous insufficiency of the lower limbs [3]. However, it was not until 1989 that this was described to be intestinal [4]. The etiology of colitis is highly varied and can include heart failure, emboli, coagulopathies, infections, medical drugs, and physical trauma. However, unlike other types of colitis, phlebosclerotic colitis is not caused by any of these [1]. Here, we present a case of a non-Asian Spanish patient who was diagnosed with phlebosclerotic colitis of submucosal veins, who was a smoker, had a history of alcohol abuse, and presented a mild factor XII deficiency. To our knowledge, this is the first described case in Spain in the literature, and only the second in Europe [2].

## Case presentation

A 62-year-old non-Asian Spanish man was admitted to the emergency room for abdominal pain of epigastric origin, which evolved over the next 3 days to diffuse pain with constipation, but with no fever or vomiting. He had a 32-pack-year smoking history and had completed disulfiram treatment for alcohol cessation 10 years previously. He had residual pain in lower limbs after lumbar disc herniation surgery 15 years previously, for which he was taking naproxen, and had a medical history of a mild factor XII deficiency. He had no other relevant medical, surgical, or family history. On physical examination, palpation showed he had a contracted abdomen, with lividity and signs of peritonism but with no signs of deep vein thrombosis in the lower limbs. Blood pressure was 112/88 mmHg, heart rate 122 beats per minute, and temperature 35.8 ℃. There were no other findings on physical and neurological examination. A thoracoabdominal computed tomography (CT) scan revealed “findings suggestive of intestinal perforation (probably in the upper hemiabdomen), without being able to clearly identify the point of perforation.” Laboratory parameters in the emergency room showed glucose 123 mg/dL, urea 238 mg/dL, creatinine 4.29 mg/dL (previously, normal), total bilirubin 1.45 mg/dL (conjugated 0.91 mg/dL), and amylase 111 mg/dL. Sodium and potassium were in normal range, and the hemogram was also normal. Intravenous fluid resuscitation and empiric antimicrobial therapy with piperacillin–tazobactam 3.375 g/8 hours intravenous for 10 days, fluconazole 400 mg/24 hours for 6 days, and vancomycin 1 g/12 hours for 4 days were started. The patient underwent urgent surgical intervention, which revealed generalized peritonitis, perforation at the pylorus, necrosis of the greater omentum, and an abundance of free fluid in all abdominal quadrants (surgical intervention consisted of pyloric suture and resection of the greater omentum). The pathology report revealed “pyloric wall with signs of perforation and epiplon with adiponecrosis, microabscesses and exudative-fibrinoid serositis.” In the immediate postoperative period, the patient had a low level of consciousness and saturation of up to 80%, with acute lung edema observed on the CT scan (“extensive bilateral pulmonary consolidations associated with bilateral pleural effusion and evidence of anasarca due to probable fluid overload/decompensated heart failure”). The patient was intubated to achieve oxygen saturation > 98% with an FiO_2_ of 0.4. The patient was sedated and on analgesic, responding to painful stimuli. Hemodynamically, he was mostly stable with a need for norepinephrine at 0.3 mg/kg/minute and tension readings of 123/73 and 90 beats per minute. However, liver function progressively worsened, reaching total bilirubin levels of 4.15 mg/dL. Likewise, the patient was in acute renal failure AKIN III, with diuresis dependent on furosemide at 30 mg/hour, creatinine levels > 4 mg/dL, and urea > 290 mg/dL. The patient presented poor postoperative course, with progressively higher fluid outflow through abdominal drains and abdominal sepsis, as revealed by symptoms and microbiology (*Klebsiella pneumoniae* was isolated in blood culture; *Enterococcus faecalis* and *Escherichia coli* in the drains; *E. coli* and *Streptococcus anginosus* in the surgical wound), which raised suspicion of perforation of the previous surgical suture. As intraabdominal pressure increased progressively, the patient underwent a second surgical intervention at 9 days after the first surgery, which revealed a biliopurulent contamination in the peritoneal cavity caused by a previous dehiscence from a pyloric suture line. Therefore, based on intraoperative findings, duodenal exclusion was performed by gastric antrectomy followed by retrocolic Roux-en-Y (end-to-side) gastrojejunostomy and jejunojejunal distal (side-to-side) anastomosis. Cholecystectomy was then followed by external drainage of the common bile duct by placement of a T-tube, to prevent cholecystitis. Splenectomy was performed because of intraoperative bleeding.

The patient remained intubated and sedated, maintaining good saturation. He also had hemodynamic stability without vasoactive amines. However, total bilirubin levels continued to rise (from 4.15 to 6.8 mg/dl), and the patient continued to exhibit anuria, with urea 291 mg/dl, creatinine 4.02 mg/dl; pH 7.35, HCO_3_^-^ 26.5 mmol/L, pCO_2_ 47 mmHg, and normal anion gap (AG). The patient began treatment with an infusion of caspofungin for 13 days at 50 mg/24 hours, meropenem for 21 days at 1 g/8 hours, and again with vancomycin for 6 days. As the patient again presented very poor postoperative evolution, with fecal contamination of the abdominal drainage, a third surgery was performed 4 days after the second one, in which the patient underwent partial colectomy (due to the intraoperative ischemic appearance of the colon) and resuture of the pyloric repair. Pathological anatomy showed gastric and duodenal dehiscence, with an ischemic appearance of the transverse colon and perforations, foci of hemorrhagic necrosis (both mucosal and submucosal), secondary gangrenous inflammation, and multiple intestinal ulcerations related to calcified phlebosclerosis of the submucosal vessels. After the third intervention, the patient was in stable but serious condition. Hemodynamically, the patient was stable, with some hypertensive peaks (160/70 mmHg). Bilirubin levels improved, down to 2.19 mg/dl; however, the patient continued to have poor kidney function that required hemodialysis sessions at 6 days after the third surgery. Due to the improvement in respiratory function, extubation was carried out but failed due to weakness in the musculature. For this reason, a tracheostomy was performed at 29 days after admission. Teicoplanin was delivered at 3 days after the third surgery, at 400 mg/12 hours for 6 days by isolation in surgical wound exudate of *Enterococcus gallinarum*. An infusion of ertapenem and ampicillin at 1 g/24 hours and 1 g/6 hours, respectively, was started at 16 days after the third surgery but was suspended because of a skin rash. Linezolid infusion (600 mg/12 hours) was also started at 20  days after the last intervention in order to treat for Gram-positive bacteria. In the last days of life, the patient showed increased overall deterioration, with disconnection to his environment, oscillation between normothermia and hypothermia, and systolic blood pressure < 70 mmHg, which forced hemodialysis to be suspended. Furthermore, despite hemodialysis, creatinine peaks continued at 8.11 mg/dl, and urea at 190 mg/dl. After receiving palliative care, the patient died, 35 days after admission. No autopsy was requested.

## Discussion and conclusions

Phlebosclerotic colitis is an extremely rare condition in Europe: it occurs almost exclusively in patients of Asian descent and is practically an unknown disease in Spain, with only one European case described to date in the literature [2]. The current case exemplifies this rarity very well: a patient without Asian descent who was admitted to the hospital because of a perforation secondary to sclerosis and calcification exclusively of the submucosal veins of the colon (phlebosclerotic colitis) who died after a torpid evolution.

The disease etiology is unknown. In 2014, Fang *et al*. [5] suggested that factors that lead to an increase in intraluminal pressure also lead to both compromised capillaries and a retention of toxic material in the colon (and especially on the right side, which has a longer retention time), which would increase their absorption and produces sclerosis, fibrosis, calcification, and hyalinization of the mesenteric and submucosal colic veins [5, 6].

**Fig. 1:**
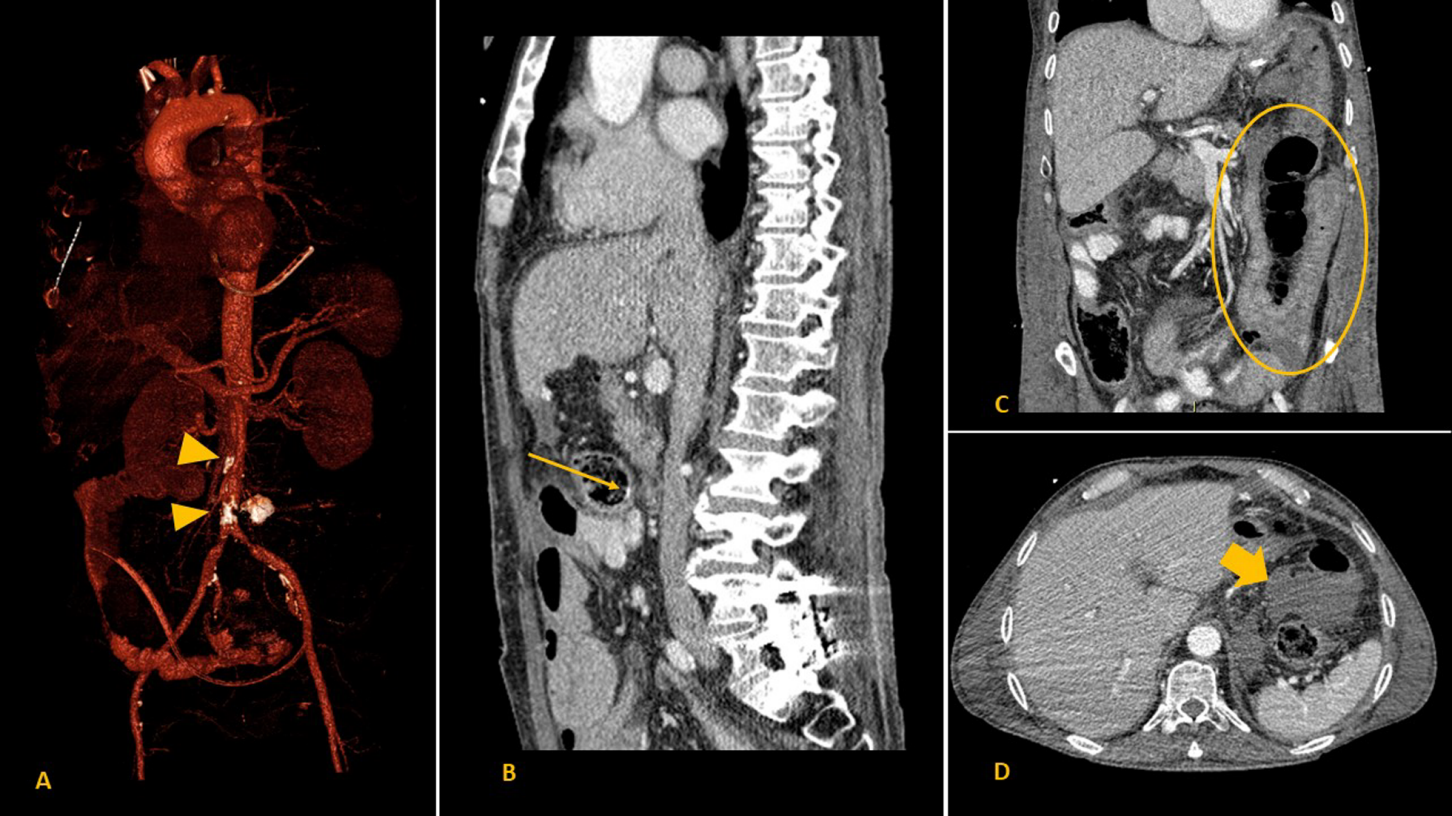
**A**–**D** Contrast-enhanced computed tomography (CECT) of the patient. **A** Three-dimensional (3D) vascular reconstruction that shows multiple atherosclerotic calcifications (triangles) but no severe stenosis in the main abdominal branches of the aorta. The CECT also displayed permeability of all veins. **B** Sagittal view of CECT, showing nonspecific linear hyperdensities of the colonic wall that could be related to incipient calcifications (arrow). **C** Coronal view of CECT, showing features of small bowel ischemia affecting the jejunum (circle), which led to a diagnosis of nonocclusive mesenteric ischemia. No ischemic changes in the colon were identified. **D** Axial view of CECT, showing that a moderate amount of intraperitoneal fluid was also present (thick arrow)

Phlebosclerotic colitis presents with nonspecific symptoms (abdominal pain, diarrhea, intestinal obstruction, nausea, and vomiting are the most frequent) and can be either acute or chronic [7, 8]. While imaging tests are essential for its diagnosis, there must also be a high level of suspicion on the part of the physicians, as the alterations can easily go unnoticed, especially in early and mild stages. CT scans typically show a thickening of the colic wall together with punctate or serpiginous calcifications of the mesenteric veins, arranged perpendicular to the longitudinal axis of the colon [9, 10]. However, the absence of these findings does not rule out the diagnosis, especially in our case, as in addition to having few calcifications at the radiological level, the patient had an exclusive submucosal involvement that did not involve mesenteric vessels (Fig. 1) [6]. Angiography in the arterial phase shows a tortuous vasa recta and the marginal arteries of the colon; in the venous phase, it shows a decreased perfusion, although this is difficult to quantify [11]. Endoscopically, a purplish and friable colic mucosa with segmental distribution is observed, with erosions, ulcers, and hemorrhagic nodules, which can be seen using a barium enema for digital imaging [12]. However, this last diagnostic test could contribute to the worsening of the disease in the acute phase due to an increase in intracolic pressure [7].

**Fig. 2:**
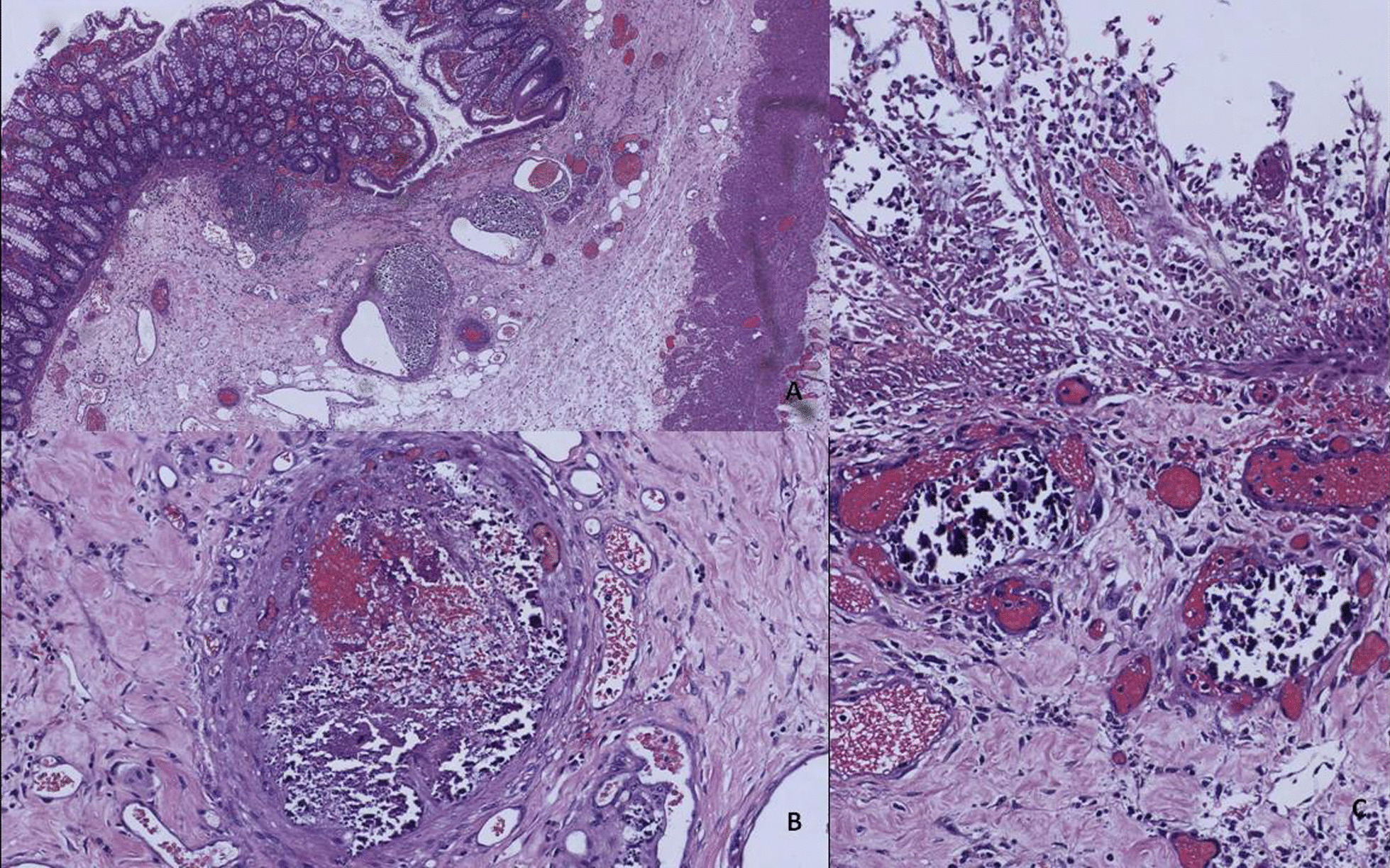
Hematoxylin and eosin (H&E)-stained tissue findings of the colon. **A** H&E image (at 4×) in which several submucosal vessels can be observed, with an important eccentric parietal calcification and a mucous ulceration that is already reepithelialized. **B** Detail (at 20×) of intense parietal calcification of a colonic submucosal vessels. **C** Magnification (10×) of images, showing intense epithelial denudation caused by vascular ischemia

Histological examinations can reveal mucosal ulceration, with foci of hemorrhagic necrosis in both mucosal and submucosal layers. The submucosal layer can show thickening due to fibrosis and hyalinization, with sclerosis, calcification, and luminal narrowing of the mesenteric and submucosal veins (the arteries are not affected). In our case, the only vessel alterations were observed in the submucosal vessels (Fig. 2). Note that mucosal atrophy and inflammation are also frequent histological findings [13].

Disease treatment varies depending on each patient's condition. Less severe conditions can be treated conservatively with follow-up, while patients with severe conditions (e.g., perforation, bleeding, and persistent intestinal obstruction) or patients whose symptoms do not remit with treatment require surgery (either subtotal or total colectomy), with a relatively good prognosis [11, 14]. In our case, given the severity and negative evolution of the patient's condition, surgery was fully indicated.

In summary, an extensive differential diagnosis is required for phlebosclerotic colitis, especially to distinguish it from other infrequent pathologies, such as idiopathic myointimal hyperplasia of the mesenteric veins, Buerger’s disease (thromboangiitis obliterans with involvement of the mesenteric vasculature), enterocolic lymphocytic phlebitis, and some types of vasculitis, such as allergic granulomatous angiitis or Behcet’s disease [13, 15]. In the case presented here, colitis could have been associated with nonsteroidal antiinflammatory drugs (NSAIDs) (given the chronic intake of naproxen); however, there was not an increased number of epithelial apoptotic bodies. Microbiological isolation of several bacteria (but not *Clostridioides difficile* or *E. coli* O157: H7) and antibiotic treatment (such as ampicillin) make it necessary to rule out pseudomembranous colitis, though pseudomembranes and fibrin thrombi were not observed (note that the submucosal veins showed calcification and sclerosis). Furthermore, the patient was treated with vancomycin. The colon was affected but with skip areas; however, we did not observe histological findings, such as lymphoid aggregates, transmural involvement, or poorly formed granulomas typical of Crohn’s disease. Finally, the differential diagnosis with calcifying uremic arteriolopathy could also be proposed, because of the similarity to the histopathological findings, but it does not fit with the previous normal kidney function and the clinical characteristics of the patient (absence of diabetes, hyperphosphatemia, obesity, dialysis, hypercoagulative states, hypoalbuminemia, dialysis, or dermal alterations).

Phlebosclerotic colitis is already a very rare disease, and its submucosal involvement even more so, with only one case reported to date. It occurs almost exclusively in people of Asian ethnicity. Due to the nonspecific symptoms and insidious radiological findings in early and mild stages as well as involvement only of the submucosal veins, it is necessary that the physician has a high suspicion to diagnose it. Angiographic and CT images show very characteristic findings of the disease; together with the endoscopic and pathological findings, they define this pathology, which has a poor prognosis. Given the low prevalence of this condition, further investigations are necessary to better understand the pathogenesis, as it is not yet clear.

## Data Availability

Not applicable. All data are included in the article.
